# Purification and identification of trichloroethylene induced proteins from *Stenotrophomonas maltophilia* PM102 by immuno-affinity-chromatography and MALDI-TOF Mass spectrometry

**DOI:** 10.1186/2193-1801-2-207

**Published:** 2013-05-07

**Authors:** Piyali Mukherjee, Pranab Roy

**Affiliations:** Department of Biotechnology, Burdwan University, Golapbag More, Burdwan, West Bengal 713104 India

**Keywords:** *Stenotrophomonas maltophilia* PM102, trichloroethylene (TCE), preadsorbed antibody, MALDI-TOF-MS

## Abstract

A novel bacterial isolate capable of growth on trichloroethylene as the sole carbon source was identified as *Stenotrophomonas maltophilia* PM102 by 16S rDNA sequencing (GenBank Acc.no. JQ797560). Serum was obtained from a rabbit immunized with the total protein extracted from the PM102 isolate grown in 0.2% TCE with 0.2% peptone. Antibodies to the common antigens were removed by preadsorbing the serum antibody on total protein extracted from the PM102 strain grown in 0.2% peptone. Western blot with the preadsorbed antibody reacted to a single band in TCE and TCE with peptone lane. No reaction was seen in peptone lane. This preadsorbed antibody specific for TCE inducible antigens was immobilised on epoxy activated sepharose 6B and total protein from PM102 cells grown in minimal medium with TCE as the sole carbon source was purified through the column. The bound protein fraction was eluted and resolved through 12% SDS PAGE. Four prominent bands observed in the protein profile were analysed by matrix-assisted laser desorption/ionization time of flight-mass spectrometry (MS) and tandem mass spectrometry (MS/MS) after in gel digestion with 25 ng/μl trypsin. A number of mono/di-oxygenases that cometabolise TCE in presence of some other primary carbon source are present in literature but this is the first attempt in identification of TCE induced proteins linked to metabolic activity with oxidoreductase like function, from a bacterial isolate that utilises TCE as the sole carbon source.

## Introduction

The health effects of Trichloroethylene are a complete adversary to its sweet odour and taste (Gist & Burg 1995). TCE arrived on the scene as an anaesthetic, but lost its medical pedigree in the 1970s when it was discovered inhaling TCE was toxic. TCE was also used in extracting caffeine from coffee beans but lost its position in the coffee industry after research uncovered its true identity as a carcinogen (Brüning et al. 1997). In homes, TCE may be found in typewriter correction fluid, paint, spot removers, carpet-cleaning fluids, metal cleaners, and varnishes. TCE is widely used in the industry as an organic solvent that can cut through grease, wax, gunk, and even silicones. Most TCE in air comes from metal degreasing activities associated with tool and automobile production. TCE enters ground water and surface water from industrial discharges or from improper disposal of industrial wastes at landfills resulting in contamination of drinking water supplies (Fan 1988;Bove et al. 1995). Therefore, extensive efforts have been made to document the biodegradation of TCE by bacteria. Although many microbes have been reported to cometabolise TCE in presence of primary carbon sources like methane (Shigematsu et al. 1999), ammonia (Arciero et al. 1989), propane (Wackett et al. 1989), etc.; we were the first to report TCE degradation activity in a *Stenotrophomonas* sp. (PM 102 isolate) that grows on TCE as the sole carbon source (Mukherjee & Roy 2012).

Studies involving identification of the proteins employed by the PM102 strain in the degradation of TCE necessitated the extraction of these proteins in a highly purified form. Immunoaffinity chromatography is one of the most powerful fractionation steps available for protein purification. Purification techniques based on the binding affinity of antigens to specific antibody have rapidly evolved using a variety of biological and synthetic ligands (Hamman & Calton 1985). The principle of protein identification using peptide mass fingerprinting (PMF) is based on the comparison of a set of experimental peptide masses obtained by trypsin digestion, with a database containing in silico digested peptide masses of known proteins (Thiede et al. 2005;Pappin et al. 1993). If the unknown protein is present in the database, match to the correct entry is obtained but if the database does not contain the unknown protein, database entries which provide closest match to equivalent proteins are chosen. Additionally, tryptic peptides can also be subjected to tandem mass spectrometry (MS/MS) where selected peptides can be further fragmented to produce a ladder of peptides for amino acid sequencing to allow high-throughput identification or confirmation of the PMF-based identification. Till date no information is available at the molecular level on proteomic compositions of bacteria capable of growth on TCE as the sole carbon source. Thus we employed immunoproteomics technology to purify and identify the TCE induced proteins from the PM102 isolate that utilizes TCE as the primary carbon source.

## Materials and methods

### Strain and growth conditions

*Stenotrophomonas maltophilia* PM102 was isolated from soil samples obtained from Asansol and Dhanbad industrial belt and identified by 16S rDNA sequencing (GenBank Acc no. JQ797560) in our laboratory. Novel TCE degradation genes were also isolated from the PM102 isolate and cloned in natural soil bacteria (Mukherjee & Roy 2013). The isolate was grown in minimal medium with 0.2% TCE, pH 6 for protein extraction and purification experiments. The minimal medium composition was (g/l): KH2PO4: 3; Na2HPO4: 6; NaCl: 0.5; NH4Cl: 1; MgSO4.7H2O: 0.5; CaCl2: 0.05.

### Fujiwara test to confirm TCE degradation by PM102 isolate

In the Fujiwara assay, polychlorinated hydrocarbons, in presence of alkali and pyridine gives a red coloured compound (Moss & Rylance 1966).

PM102 cells were grown in 50 ml minimal medium with 0.3% TCE, at pH5 and pH 7 respectively. 2 ml aliquot was taken at the beginning (just after inoculation) and after every 30 minutes interval upto 120 minutes and treated with 2 ml 5 N NaOH and 2 ml pyridine followed by heating at 80°C for two minutes. Absorbance of the upper red phase was recorded at 470 nm by spectrophotometer. A control was set up with *E.coli* in LB medium with 0.3% TCE. A standard curve was plotted by varying TCE concentration from 0.01% to 0.48%, from which the amount of TCE remaining at each time point was calculated.

### Protein extraction

The PM102 cells from 50 ml culture was harvested by centrifugation at 4°C, 10,000 rpm for 10 minutes. Cell pellet thus obtained was suspended in 1 ml solution 1 (10 mM EDTA pH8, 50 mM glucose, 25 mM Tris–HCl pH8) with 100 μl of 10 mg/ml lysozyme, vortexed and incubated at 37°C for 1 hr followed by 30 minutes incubation at 4°C. Lysozyme extraction gave better cell lysis when followed by temperature shock. The suspension was centrifuged at 10,000 rpm for 10 minutes at 4°C and the pellet containing cell debris was discarded. Supernatant thus obtained containing the intracellular proteins was stored at −20°C. Concentration of proteins was determined by Bradford assay (Bradford 1976).

### Generation of specific antibody

A rabbit was injected subcutaneously at 4 to 5 different sites with total protein isolated from the PM102 strain grown in minimal medium with 0.2% TCE and 0.2% peptone, mixed 1:1 with Freund’s complete adjuvant. The injections were given once per month for two consecutive months followed by two more injections of the same protein mixed 1:1 with Freund’s incomplete adjuvant. The serum antibody thus obtained was preadsorbed on peptone grown cellular proteins. Small strips of nitrocellulose membrane were soaked in total protein isolated from PM102 cells grown in 0.2% peptone and air dried. These strips were immersed in the serum antibody diluted 1:100 with buffer A (10 mM tris HCl pH8, 1 mM EDTA pH8, 0.05% tween 20 and 0.9% NaCl) under mild shaking for 1 hour. This process was repeated thrice until all antibodies to the common peptone antigens were removed. The antibody solution finally obtained was specific against TCE inducible proteins of the PM102 isolate.

### Dot blot to check antibody specificity

Two strips of nylon membrane were spotted with 3 μl of proteins extracted from *Stenotrophomonas maltophilia* PM102 grown in minimal medium with 0.2% TCE as the sole carbon source and 3 μl of 1 mg/ml BSA. The spots were air dried and membranes were blocked with 3% milk powder in buffer A (10 mM tris HCl pH8, 1 mM EDTA pH8, 0.05% tween 20 and 0.9% NaCl) for 20 minutes in rocker. The membranes were washed thrice in buffer A. One membrane was incubated in pre-immunised serum (1:100 dilutions in buffer A) while the other membrane was incubated in immunised serum (1:100 dilutions in bufferA) for 1 hour in rocker followed by washing in buffer A. The membranes were incubated in (1:15,000 dilution in bufferA) of Goat antirabbit IgG coupled to alkaline phosphatase (Sigma Aldrich USA) for 30 minutes in rocker. After washing with buffer A thrice, the membranes were equilibriated in alkaline phosphatase buffer (100 mM tris HCl pH 9.5, 100 mM NaCl and 5 mM MgCl_2_) and developed with BCIP/NBT (5-Bromo,4-Chloro,3-Indolyl phosphate/Nitrobluetetrazolium).

### Western blot

Proteins extracted from *Stenotrophomonas maltophilia* PM102 grown in minimal medium with 0.2% peptone, 0.2%TCE with 0.2% peptone and 0.2%TCE sole were resolved by 12% SDS-PAGE. The SDS PAGE gel, after electrophoresis (unstained) was electro-blotted onto nitrocellulose membrane (Sigma Aldrich USA) at 45 volts for 3 hours. The next steps were same as in dot blot: the nitrocellulose membrane was blocked in 3% milk powder and incubated in serum antibody overnight at 4°C followed by incubation in secondary antibody (1:15,000 dilutions in buffer A) of Goat antirabbit IgG coupled to alkaline phosphatase. Each step was followed by thorough washing the membrane in buffer A. Finally, the membrane was incubated in alkaline phosphate buffer and developed with BCIP/NBT. Immuno-blotting was carried out with total serum antibody as well as preadsorbed serum antibody.

### Immuno affinity column chromatography

2gm epoxy activated sepharose 6B (Sigma Aldrich, USA) was suspended in 10 ml 1 M tris pH8 overnight. The sepharose was packed in a 10 ml syringe (used as column). As high salt and high pH facilitate binding, 10 ml binding buffer (1 N KH2PO4, pH9) was recycled through the column thrice. Flow rate was maintained at 50 drops/minute. The preadsorbed antibody solution was loaded in the column and allowed to immobilise. 1 ml of the total protein extracted from the PM102 strain grown with TCE as the sole carbon source was added to the column. Unbound antigen fraction was collected and the column was washed with wash buffer (phosphate buffer saline pH7.4 with 1% tween20) to remove remaining unbound antigens. Protein fraction bound to the immobilised antibody was collected in elution buffer (0.1 M glycine, pH2.8). The antibody was recovered in regeneration buffer (0.1 M glycine, pH2.4).

### Protein profile and image analysis

The unbound and bound protein fractions were resolved by 12% SDS PAGE and stained with coomassie blue. The coomassie stained gel was analysed with the image analysis tool of Quantum Capt software in the gel documentation system (Vilbur Lourmat, France) to predict the molecular weights of the purified protein bands.

### In-gel digestion

The four purified protein bands: P1, P2, P3 and P4 were excised from the gel and placed in separate eppendroff tubes prewashed with 500 μl 0.1% trifluoroacetic acid (TFA)/60% acetonitrile (HPLC grade). 250 μl freshly prepared 100 mM ammonium bicarbonate/acetonitrile (1:1, v/v) was added to the tubes kept at 37°C for 30 minutes with occasional vortexing to destain the gel pieces. The solution was discarded carefully and 250 μl 20 Mm ammonium bicarbonate/acetonitrile (1:1, v/v) was added for further destaining. Next, the gel pieces were immersed in 500 μl acetonitrile for 15 minutes till they shrank and became white in colour. The acetonitrile was pipetted out and gel pieces were air dried at room temperature for 10 minutes. The dried gel pieces were reswollen in 20 μl 25 ng/μl trypsin (mass spectrometry grade, Sigma Aldrich, USA). Initially, trypsin digestion was done at 4°C for 2 hours followed by overnight digestion at 37°C. If the trypsin solution dried up, 2 to 3 drops of 50 mM ammonium bicarbonate was added. The trypsin digested protein solutions were taken in fresh tubes and lyophilised for 1 hour. The lyophilised product was dissolved in 5 μl 30% acetonitrile with 0.1% TFA. 4.5 μl of the concentrated peptide solutions were spotted on the target plate with 4.5 μl CHCA matrix solution.

### MALDI-TOF-MS and MS/MS analysis

Mass spectra were obtained in a MALDI-TOF mass spectrometer equipped with Applied Biosystems 4700 Proteomics Discovery System (Darmstadt, Germany), which employs novel results dependent analysis features to trigger MS/MS analysis of peptides based on single-stage MS peptide mass fingerprinting (PMF) results. It has the GPS Explorer™ software that provides features to remotely submit acquisition jobs to the 4700 Proteomics Analyzer. RDA™ software was used to specify the confidence interval for protein identification and the number of matching or non-matching peptides to acquire MS/MS spectra. Database search was performed with industry standard MASCOT database search engine (Perkins et al. 1999).

## Results

### TCE degradation assay

**Figure 1:**
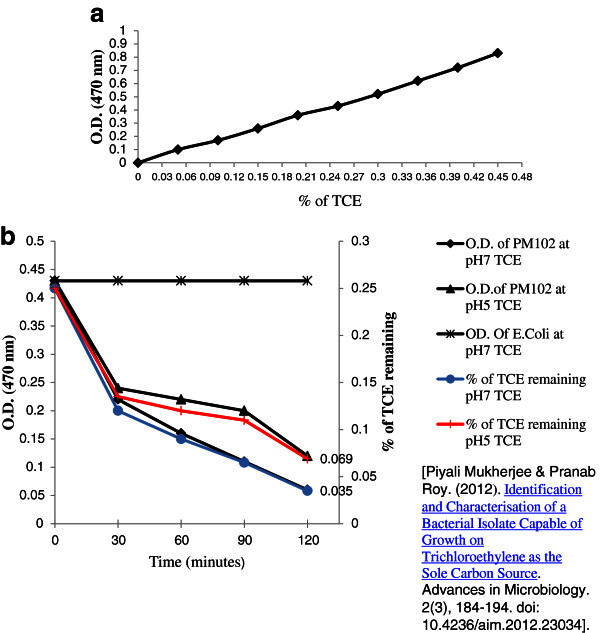
**Fujiwara test to confirm TCE degradation by the PM102 isolate.a**. Standard curve of% of TCE plotted against absorbance. **b**. Plot showing the decrease in absorbance corresponding to the catabolism of 0.3% TCE added initially, by PM102, measured at pH 5 and pH 7 respectively. Secondary vertical axis shows the% of TCE remaining at different time intervals as calculated from the standard curve.

The Fujiwara test for TCE degradation by PM102, showed a decrease in absorbance corresponding to decrease in colour intensity of the upper phase with time, thus confirming TCE degradation. The control setup with *E.coli* gave a parallel graph indicating no change in colour. The percentage of TCE remaining after 120 minutes at pH 7 was 0.035% while at pH 5 was 0.069%, of the 0.3% added initially. (Figure 1a and b). From this TCE disappearance assay, it was calculated that 90% TCE was degraded at pH7 while 77% TCE was degraded at pH 5 under conditions where TCE was the only carbon source.

### Dot blot

**Figure 2:**
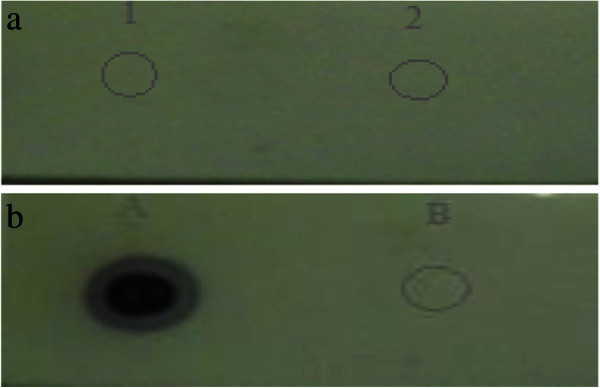
**a. Dot blot with pre-immunised serum obtained from rabbit.** Spot 1: 3 μl of proteins extracted from PM102 isolate grown in 0.2% TCE as the sole carbon source. Spot 2: 3 μl of 1 mg/ml BSA. **b.** Dot blot with immunised serum antibody. Spot A: 3 μl of proteins extracted from PM102 isolate grown in 0.2% TCE as the sole carbon source. Spot B: 3 μl of 1 mg/ml BSA.

It was observed that the pre-immunised serum did not react with PM102 proteins whereas reaction was obtained with immunised serum antibody against proteins extracted from PM102 isolate. Also no reaction was seen against BSA with both the immunised and pre-immunised serum. Figure 2a and b show the results obtained in dot blot assay.

### Western blot

**Figure 3:**
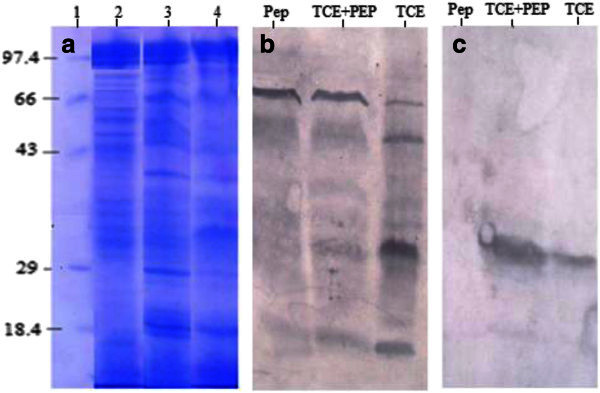
**Successful antibody subtraction shown by Western Blot and corresponding SDS PAGE profile.a**. 12% SDS-PAGE showing proteins extracted from the PM102 isolate. Lane1: medium molecular weight marker; Lane2: 0.2% peptone; Lane3: 0.2%TCE + 0.2% peptone; Lane4: 0.2% TCE. **b**. Western blot with total serum antibody was found to recognise many proteins. **c**. Western blot with preadsorbed serum antibody recognised a single protein band of 35.14 kDa in TCE lane and TCE + peptone lane. No reaction seen in peptone lane.

Figure 3a shows the 12% SDS-PAGE profile of proteins extracted from the PM102 isolate with different substrates as the primary carbon source: peptone, TCE and TCE + peptone. When this gel was electro-blotted on nitrocellulose membrane and incubated in total serum antibody, many bands were seen in response to PM102 proteins but immuno-blot carried out with the preadsorbed serum antibody reacted against a single protein band of 35.14 kDa.

### Profiling of immuno-affinity column purified proteins

**Figure 4:**
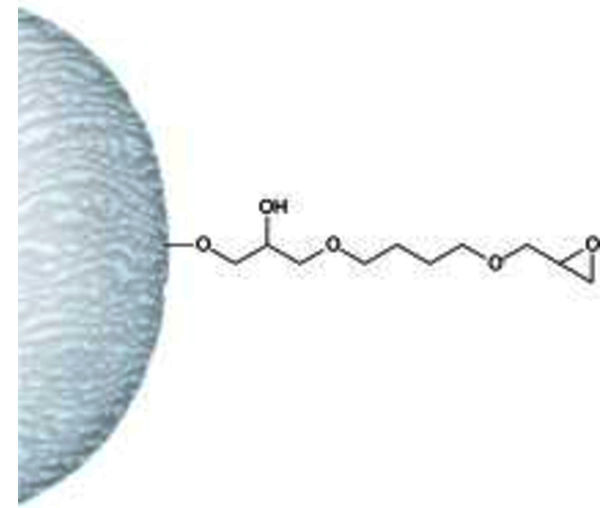
**Epoxy activated sepharose 6B with 12 atom spacer arm the binds to CHO group via stable ether to hydroxyl linkages.**

Epoxy activated sepharose 6B, formed by reacting Sepharose 6B with 1,4-bis (2,3- epoxy-propoxy-) butane couples carbohydrates via stable ether linkages to hydroxyl groups through a 12 atom hydrophilic spacer arm (Figure 4). Antibodies get covalently attached to sepharose 6 B via the carbohydrate (CHO) residues in their Fc region (Subramanian 2000).

**Figure 5:**
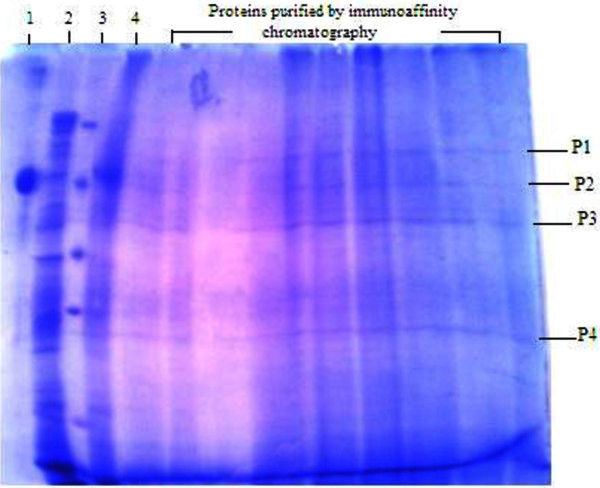
**12% SDS PAGE of TCE induced proteins from PM102 isolate purified through immuno-affinity column chromatography.** Lane 1- BSA, Lane 2- total crude protein extracted from PM102 cells grown with 0.2% TCE as the sole carbon source, Lane 3- medium molecular weight marker of 97, 66, 43, 29 and 18.4 kDa respectively, Lane 4 – unbound antigen, lanes 5 to13– bound protein fraction. Four distinct protein bands: P1, P2, P3 and P4 were observed after immuno-affinity column purification of 0.2% TCE grown proteins of PM102 isolate.

12% SDS PAGE carried out with the immuno-purified bound protein fraction gave four distinct protein bands. Analysis of the SDS PAGE with Quantum Capt software revealed the molecular weights of the four protein bands designated P1, P2, P3 and P4 to be: 68 kDa, 59 kDa, 46 kDa and 24 kDa respectively (Figure 5).

### Identification of the purified protein bands

**Figure 6:**
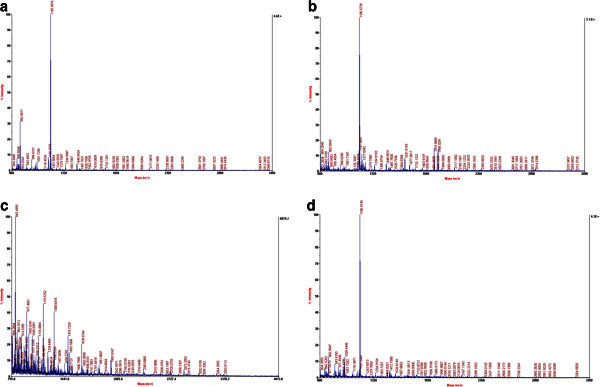
**Mass spectrum of the proteins purified through immuno-affinity column chromatography obtained after in gel digestion with 25 ng/l Trypsin.a**. Mass spectrum of immuno-purified protein band: P1. **b**. Mass spectrum of immuno-purified band: P2. **c**. Mass spectrum of immuno-purified protein band: P3. **d**. Mass spectrum of immuno-purified protein band: P4.

**Figure 7:**
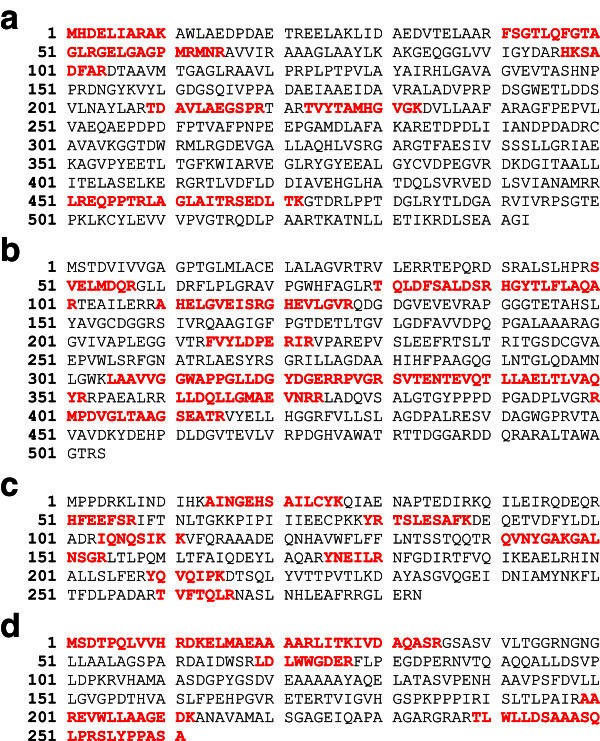
**Peptide match of the four immune-purified protein bands to their respective top hits obtained through MASCOT database search.a**. Immuno-purified protein P1matched peptides to putative phosphor-mannomutase from *Streptomyces avermitilis* MA-4680 (gi|29606994) as observed through MASCOT database search with Applied Biosystems 4700 Proteomics Analyser. Matched peptides shown in bold red. **b**. Protein P2 matched to a putative monooxygenase from *Streptomyces coelicolor* A3 (gi|5763908). **c**. Protein P3 matched to a hypothetical protein from *Bacillus licheniformis* (Q65KM5_BACLI). **d**. Protein P4 closest match was to a putative 6-phosphogluconolactonase from *Streptomyces coelicolor* A3 (gi|5459406).

Mass spectra of the immuno-purified proteins from PM102 isolate are shown in Figure 6a, b, c, d. The MASCOT search results gave the closest match to the purified proteins as present in the database. P1 was matched to a putative phosphormannomutase from *Streptomyces avermitilis* MA-4680 with accession number: (gi|29606994) (Figure 7a); P2 closest match was a putative monooxygenase from *Streptomyces coelicolor* A3 (gi|5763908) (Figure 7b); P3 was matched to a hypothetical protein from *Bacillus licheniformis.*(Q65KM5_BACLI) (Figure 7c) and P4 closest match was a putative 6-phosphogluconolactonase from *Streptomyces coelicolor* A3 (gi|5459406) (Figure 7d). The protein accession numbers obtained through MASCOT were searched in the Universal Protein Resource (Uniprot) database and available literature for assigning the functional groups are presented in Table 1.

**Table 1:** **Identification of the immuno-purified proteins from PM102 isolate that grows on TCE as the sole carbon source and functional assignment observed through MASCOT database search**

Protein band	Protein identified	Accession number	Protein function	Probability based score	Nominal mass (Da).	Calculated pI value	Sequence coverage
P1	Putative Phosphomannomutase from *Streptomyces avermitilis* MA-4680	gi|29606994	Carbohydrate metabolism, Mg ion binding.	47	58132	5.01	16%
		(Q82I15_STRAW)					
P2	Putative monooxygenase from *Streptomyces coelicolor* A3	(gi|5763908)	Oxido reductase activity	57	53859	5.66	26%
		(Q9S1P2_STRCO)					
P3	Hypothetical protein from *Bacillus licheniformis*	(Q65KM5_BACLI)	Oxido reductase activity	69	32917	6.62	29%
P4	6-phospho gluconolactonase from *Streptomyces coelicolor* A3	(gi|5459406)	Catalyses 2^nd^ step of oxidative phase of the pentose phosphate pathway.	54	27194	5.38	31%
		Q9XAB7 (6PGL_STRCO)					

Cell-PLoc: A package of web-servers for predicting subcellular localization of proteins in various organisms (Chou & Shen 2008) was used to find the location of the identified proteins. P1and P4 was found to be located in membrane bound form while P2 and P3 were located in the cytoplasm.

## Discussion

Many protein bands were obtained with the crude protein extracted from PM102 cells grown in minimal medium with 0.2% TCE as the sole carbon source but after immune purification of this crude protein, only four bands were detected in the SDS profile. The preadsorbed antibody used in the affinity chromatography based protein purification technique, was specific against TCE inducible antigens from PM102 as it was noted that no reaction was seen against proteins extracted from peptone grown PM102 cells with the preadsorbed antibody. The preadsorbed antibody reacted strongly against a single protein of 35.14 kDa indicating successful antibody subtraction. PM102 proteins purified by affinity chromatography with this preadsorbed antibody gave four bands in the gel profile indicating that although the preadsorbed antibody gave strong response against a single protein that corresponds to immuno-purified band P3, it might have weak reactions against the other three purified protein bands: P1, P2 and P4.

The four immuno-purified proteins were identified by MALDI-TOF-MS and MS/MS analysis. All the four proteins were found to be linked to metabolic activity with oxidoreductase like function. The genome of *Stenotrophomonas maltophilia* has been sequenced and is available in the databases. In the mascot search with PMF data, the top hits of the four immuno-purified proteins from *S.maltophilia* PM102 did not match to any of the *S.maltophilia* proteins in the database records. *Stenotrophomonas maltophilia* strains are found to be ubiquitously distributed in the environment with regard to habitat and geography and have been shown to play important role in bioremediation. (Binks et al. 1995;Nawaz et al. 1993;Wang et al. 1997). As a result of this adaptation to different habitats, great metabolic heterogeneity has been found (Berg et al. 1999;Hauben et al. 1999). *S. maltophilia* exhibit a high level of genetic diversity even when isolated from the same area (Valdezate et al. 2004). Due to lateral gene transfer, some closely related bacteria can have very different morphologies and metabolisms. Genetic changes in bacterial genomes come from either random mutation during replication or "stress-directed mutation", where genes involved in a particular growth-limiting process have an increased mutation rate (Wright 2004). Mutation rates vary widely among different species of bacteria and even among different clones of a single species of bacteria (Denamur & Matic 2006). This explains that the PM102 isolate may be genetically and metabolically diverse from the *S. maltophilia* strains that are reported in the databases.

To minimise peptide loss during sample preparation for in gel digestion, the peptide extraction step was omitted and 25 ng/l trypsin was used to obtain better mass spectra (Kundu et al. 2012).

Nominal mass of the proteins identified by MASCOT search with MALDI-TOF mass spectrum data were within the range of 58 kDa to 27 kDa. This pattern almost coincided with the molecular weights of the original protein bands purified with antibody specific to TCE inducible proteins from PM102 isolate predicted through Quantum Capt software. Calculated pI of these proteins were in the acidic range: 5.01 to 6.62. Protein sequences of monooxygenases and dioxygenases involved in TCE cometabolism were retrieved from Uniprot database and their theoretical molecular weights and pI were determined with Compute pI tool at Expasy Bioinformatics Resource Portal (Bjellqvist et al. 1993;Gasteiger et al. 2005). From the data thus obtained (presented in Table 2), it was found that these proteins too had molecular weights in the range 63 kDa to 22 kDa and pI ranging between 4.79 to 6.77, as seen with the TCE inducible proteins identified from PM102 strain. All these oxygenases are known to be responsible for TCE degradation in presence of some other primary carbon source like methane, propane, toluene, benzene, etc. but the oxygenases identified from the PM102 isolate were extracted and purified under conditions where TCE was available to the bacterium as the sole carbon source. Further experiments need to be done to demonstrate the exact mechanism of TCE metabolism by the four identified proteins.

**Table 2:** **Molecular weights and pI of oxygenases known to play a role in TCE cometabolism (predicted through compute pI tool at Expasy Bioinformatics Resource Portal)**

Oxygenases involved in TCE cometabolism	Uniprot accession numbers	Predicted molecular weights (Da).	Predicted pI
Toluene dioxygenase large subunit) from *Pseudomonas putida*	Q3LWS6	50944.27	5.27
Benzene dioxygenase α subunit from *P. putida*	A5W4F2	50944	5.27
Benzene dioxygenase β subunit from *P. putida*	A5W4F1	22012.81	5.78
Methane monooxygenase from *Methylosinus trichosporium*	Q53563	37991.29	5.80
Ammonia monooxygenase from *Nitrosomonas europaea*	Q04508	46792.93	6.77
Propane monooxygenase from *Rhodococcus sp*.	Q0SJK9	63222.42	5.56
Phenol hydroxylase from *Pseudomonas sp.*	P19734	38477.58	4.79

## References

[CR1] Arciero D, Vannelli T, Logan M, Hooper AB (1989). Degradation of trichloroethylene by ammonia oxidizing bacterium *Nitrosomonas europaea*. Biochem Biophys Res Commun.

[CR2] Berg G, Roskot N, Smalla K (1999). Genotypic and phenotypic relationships between clinical and environmental isolates of Stenotrophomonas maltophilia. J Clin Microbiol.

[CR3] Binks PR, Nicklin S, Bruce NC (1995). Degradation of hexahydro-1,3,5-trinitro-1,3,5-triazine (RDX) by Stenotrophomonas maltophilia PB1. Appl Environ Microbiol.

[CR4] Bjellqvist B, Hughes GJ, Pasquali C, Paquet N, Ravier F, Sanchez J-C, Frutiger S, Hochstrasser DF (1993). The focusing positions of polypeptides in immobilized pH gradients can be predicted from their amino acid sequences. Electrophoresis.

[CR5] Bove FJ, Fulcomer MC, Klotz JB, Esmart J, Dufficy EM, Savrin JE (1995). Public drinking water contamination and birth outcomes. Am J Epidemiol.

[CR6] Bradford MM (1976). A rapid and sensitive method for the quantitation of microgram quantities of protein utilizing the principle of protein-dye binding. Anal Biochem.

[CR7] Brüning T, Weirich G, Hornauer MA, Höfler H, Brauch H (1997). Renal cell carcinomas in trichloroethene (TRI) exposed persons are associated with somatic mutations in the von Hippel-Lindau (VHL) tumour suppressor gene. Arch Toxicol.

[CR8] Chou KC, Shen HB (2008). Cell-PLoc: a package of Web servers for predicting subcellular localization of proteins in various organisms (updated version: Cell-PLoc 2.0: An improved package of web-servers for predicting subcellular localization of proteins in various organisms, Natural Science, 2010, 2, 1090–1103). Nat Protoc.

[CR9] Denamur E, Matic I (2006). Evolution of mutation rates in bacteria. Mol Microbiol.

[CR10] Fan AM (1988). Trichloroethylene: water contamination and health risk assessment. Rev Environ Contam Toxicol.

[CR11] Gasteiger E, Hoogland C, Gattiker A, Duvaud S, Wilkins MR, Appel RD, Bairoch A, Walker JM (2005). Protein Identification and Analysis Tools on the ExPASy Server. The Proteomics Protocols Handbook.

[CR12] Gist GL, Burg JR (1995). Trichloroethylene — a review of the literature from a health effects perspective. Toxicol Ind Health.

[CR13] Hamman JP, Calton GJ (1985). Immunosorbent chromatography for recovery of protein products. ACS Symp. Ser.—Purification of Fermentation Products.

[CR14] Hauben L, Vauterin L, Moore ER, Hoste B, Swings J (1999). Genomic diversity of the genus Stenotrophomonas. Int J Syst Bacteriol.

[CR15] Kundu S, Chakraborty D, Das K, Pal A (2012). An efficient in-gel digestion protocol for mass spectral analysis by MALDI-TOF-MS and MS/MS and its use for proteomic analysis of vigna mungo leaves. Plant Mol Biol Rep.

[CR16] Moss MS, Rylance HJ (1966). The Fujiwara reaction: some observation on the mechanism. Nature.

[CR17] Mukherjee P, Roy P (2012). Identification and characterisation of a bacterial isolate capable of growth on trichloroethylene as the sole carbon source. Advances in Microbiology.

[CR18] Mukherjee P, Roy P (2013). Cloning, sequencing and expression of novel trichloroethylene degradation genes from *Stenotrophomonas maltophilia* PM102: a case of gene duplication. J Bioremed Biodeg.

[CR19] Nawaz MS, Franklin W, Cerniglia CE (1993). Degradation of acrylamide by immobilized cells of a Pseudomonas sp. and Xanthomonas maltophilia. Can J Microbiol.

[CR20] Pappin DJ, Hojrup P, Bleasby AJ (1993). Rapid identification of proteins by peptide-mass fingerprinting. Curr Biol.

[CR21] Perkins DN, Pappin DJC, Creasy DM, Cottrell JS (1999). Probability-based protein Identification by searching sequence databases using mass spectrometry data. Electrophoresis.

[CR22] Shigematsu T, Hanada S, Eguchi M, Kamagata Y, Kanagawa T, Kurane R (1999). Soluble methane monooxygenase gene clusters from trichloroethylene-degrading Methylomonas sp. strains and detection of methanotrophs during in situ bioremediation. Appl Environ Microbiol.

[CR23] Subramanian A (2000). Immunoaffinity chomatography.

[CR24] Thiede B, Höhenwarter W, Krah A, Mattow J, Schmid M, Schmidt F, Jungblut PR (2005). Peptide mass Fingerprinting. Methods.

[CR25] Valdezate S, Vindel A, Martín-Dávila P, Del Saz BS, Baquero F, Cantón R (2004). High genetic diversity among *Stenotrophomonas maltophilia* strains despite their originating at a single hospital. J Clin Microbiol.

[CR26] Wackett LP, Brusseau GA, Householder SR, Hanson RS (1989). Survey of microbial oxygenases: trichloroethylene degradation by propane-oxidxing bacteria. Appl Environ Microbiol.

[CR27] Wang X, Li B, Herman PL, Weeks DP (1997). A three-component enzyme system catalyzes the O Demethylation of the herbicide dicamba in Pseudomonas maltophilia DI-6. Appl Environ Microbiol.

[CR28] Wright B (2004). Stress-directed adaptive mutations and evolution. Mol Microbiol.

